# Lectotypification of the name *Stereodon nemoralis* Mitt. (Plagiotheciaceae), a basionym of *Plagiothecium nemorale* (Mitt.) A. Jaeger

**DOI:** 10.3897/phytokeys.155.51469

**Published:** 2020-08-07

**Authors:** Grzegorz J. Wolski, Anna Faltyn-Parzymska, Jarosław Proćków

**Affiliations:** 1 Department of Geobotany and Plant Ecology, Faculty of Biology and Environmental Protection, University of Lodz, ul. Banacha 12/16, 90-237 Lodz, Poland University of Lodz Lodz Poland; 2 Department of Plant Biology, Institute of Biology, Faculty of Biology and Animal Science, Wrocław University of Environmental and Life Sciences, ul. Kożuchowska 7a, 51-631 Wrocław, Poland Wrocław University of Environmental and Life Sciences Wrocław Poland

**Keywords:** Codes of Botanical Nomenclature, Hooker collection, India, Mitten collection, nomenclatural types, *
Plagiothecium
*, *
Stereodon
*, typification

## Abstract

In 1859, William Mitten described *Stereodon nemoralis* (≡ *Plagiothecium nemorale*) based on the gathering of Sir J.D. Hooker from India. However, the protologue did not indicate any specific specimen or illustration. For the past 50 years, the original material (NY 913349) deposited at the NY Herbarium has been considered as the holotype. However, this assumption has since been found to be incorrect, because in the Herbarium of The Natural History Museum exists other original material of this species (BM 1030713), collected by Hooker. In addition, the specimen from NY Herbarium is in poor condition and its most important diagnostic characters are not visible. In contrast, the material from BM Herbarium is in very good condition, and therefore it is herein designated as the lectotype. Also, the paper describes the resolution of this type, a process complicated by changes that had occurred in the provisions of subsequent botanical *Codes*.

## Introduction

During his travels around India (between 1847–1851), staying from 20 to 24 May 1848 on Mount Tonglo (alt. 3036 m) in the Singalila Range in the Eastern Himalayas of Sikkim Sir Joseph Dalton Hooker collected many specimens of mosses (Hooker 1854). Collections gathered by him were subsequently examined by W. Mitten and W. Wilson, who, based on their findings, published jointly an incomplete provisional list of moss taxa from this expedition (Mitten and Wilson 1857). In 1859 Mitten also used this collection for the publication of his famous *Musci Indiae Orientalis* in which he described many new species, including *Stereodon nemoralis* (Mitten 1859).

The first set of specimens is preserved in the Hooker’s Herbarium which was acquired in 1867 by Kew Gardens (K), but after World War II the bryophyte collection in K, thanks to a decision of the British government, was transferred to the British Museum (BM). Mitten retained many duplicates in his private herbarium which after his death in 1903 was bought by the New York Botanical Garden (NY).

*Stereodon nemoralis* was incorporated into the genus *Plagiothecium* by Augusto Jaeger in 1878, and its name changed to *Plagiothecium nemorale* (Mitt.) A. Jaeger (Jaeger 1878). Since then, many new taxa have been described within the genus; however, the number of potential species is still unclear (Wolski 2018). In the most recent revision of the genus, Wynns (2015) recognised 67 taxa, with a further 46 awaiting detailed research to determine their taxonomic status. Therefore, it can be expected that the number of species belonging to this genus will change in the near future, especially taking into account the increasing interest in research of the genus, as well as the more widespread use of molecular methods. These observations have been confirmed by recent research (Wynns 2015; Wynns and Schröck 2018; Ignatova and al. 2019; Wolski and Nowicka-Krawczyk 2020).

*Plagiothecium nemorale* has long been a neglected species, until it was resurrected from obsolescence by Iwatsuki (1970), when he found that it was the oldest available name for *P. sylvaticum* which proved to be conspecific with *P. denticulatum* (Hedw.) Schimp. and *P. neglectum* Mönk. Iwatsuki (1970) also proposed the specimen collected by J.D. Hooker, and currently deposited at the NY Herbarium (NY 913349), to accept to be the holotype of the name *P. nemorale*. In this revision of the genus, as in others, no other indicated type specimens or original material appear with the name *P. nemorale*.

## Materials and methods

After Mitten’s death in 1906, his entire herbarium was purchased by the NY Herbarium, and that is where most of the types of the species described by Mitten can currently be found. In addition, according to Thiers (1983), and information obtained from the curators of the **FH**, **G**, **MICH**, and **NY** herbaria during the conducted research, the types of the species from the *Plagiothecium* genus given by Mitten are deposited only in the **NY** Herbarium. In contrast, the **FH**, **G** and **MICH** collections only include selected duplicate specimens (but not types) from his collection.

A number of other herbaria from around the world were also searched, as well as various virtual databases with a global reach, such as JSTOR Global Plants, GBIF, Tropicos, INCT – the Virtual Herbarium of Flora and Fungi, the Chinese Virtual Herbarium and the Consortium of North American Bryophyte Herbaria (CNABH). A list of all checked herbaria is available on request directly from the first author.

## Results

**Figure 1.**
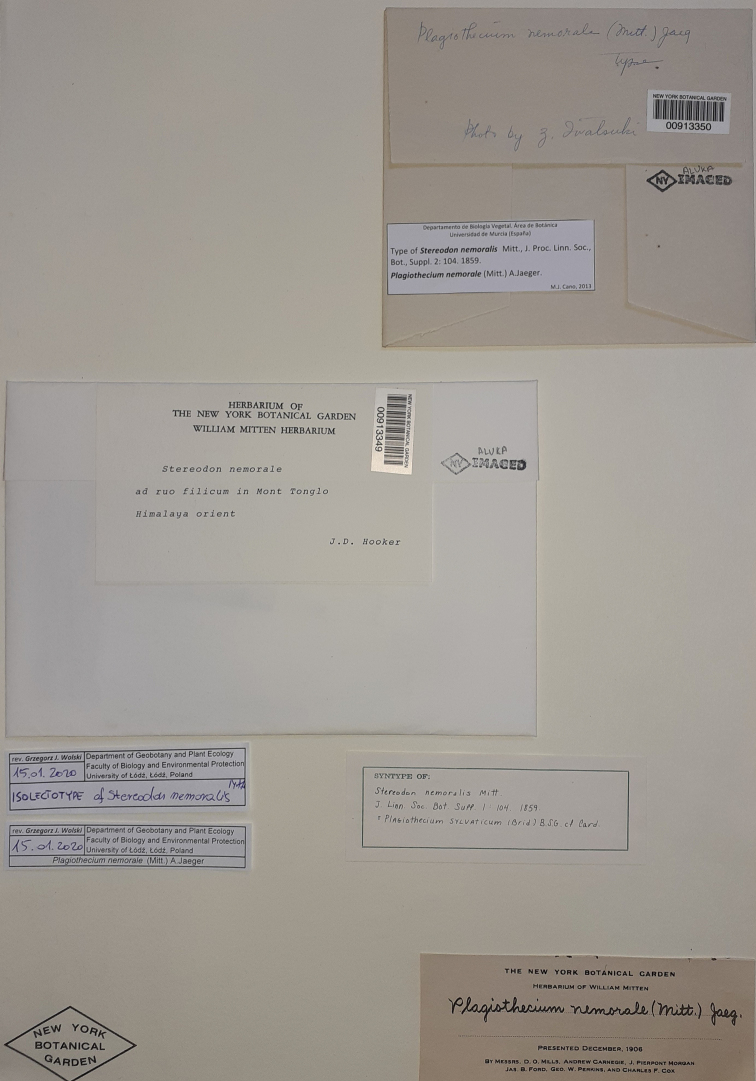
Herbarium sheet of *Stereodon nemoralis* Mitt. deposited in the New York Botanical Garden Herbarium (NY 913349 and NY 913350).

**Figure 2.**
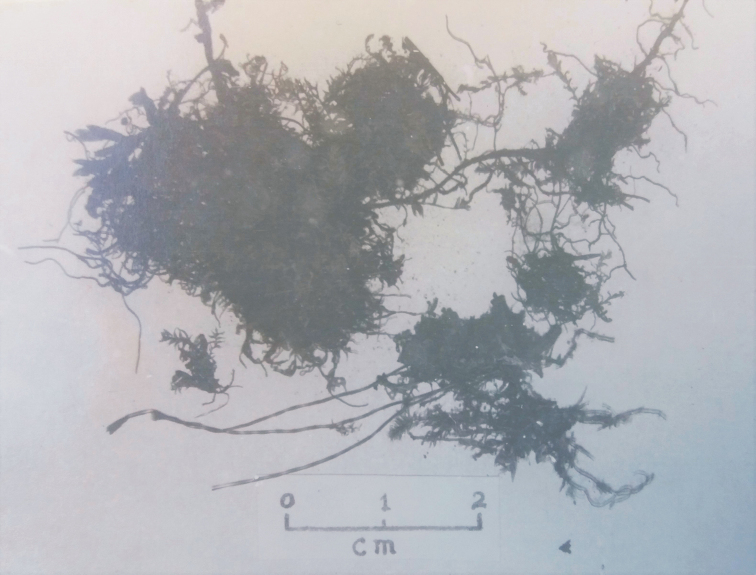
Photograph of the specimen NY 913349 taken by Iwatsuki (stored as NY 913350).

**Figure 3.**
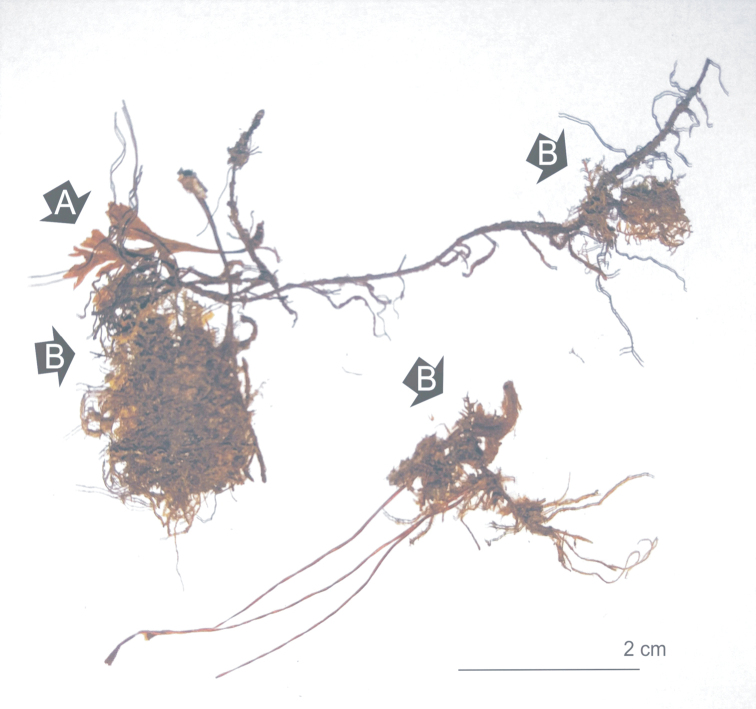
Remains of the holotype of the *Stereodon nemoralis* Mitt. (NY 913349) **A** young fern **B** stems of *Fissidens* sp. moos.

In 2016 and 2018, during the taxonomic revision of *Plagiothecium nemoralesensu lato*, its type specimens were examined. It was found that the NY Herbarium included two specimens with separate numbers on one herbarium sheet (Fig. 1). However, one of them (NY 913350) was not, in fact, a plant specimen, but a photograph of specimen NY 913349 (Fig. 2) with the note “Type. Photo by Z. Iwatsuki” (Fig. 1). Hence, NY 913350 is a photograph of plant specimen NY 913349 located on the same herbarium sheet but marked with a different number (Fig. 3).

The information given on the herbarium sheet, comprising the name of the collector, the description of the location, and even the substratum on which the specimen grew, i.e. the fern visible in the picture, is the same as in the protologue (Mitten 1859). Based on this similarity, and it being the only original known specimen, it has been recognised as a holotype by various researchers, including Iwatsuki (1970) and Thiers (1992).

The herbarium sheet also included a label with the inscription: “Syntype of: *Stereodon nemoralis* Mitt. J. Linn. Soc. Bot. Supp. 1: 104. 1859 ≡ *Plagiothecium sylvaticum* (Brid.) B.S.G. cf. Card.”. It was not possible to determine when the label had been stuck to the herbarium sheet: it could have been a few years or several decades previously. Nevertheless, it was reported to have been placed there by the NY Herbarium staff by mistake (Herbarium staff, pers. comm.). However, this appeared to be a mistake only until July 2011, when the *Vienna Code* ceased to apply (McNeill et al. 2006). From this time, when the *Melbourne Code* came into force (McNeill et al. 2012), and Recommendation 9A.4 originally introduced by the *Vienna Code*, i.e. “When a single gathering is cited in the protologue, but a particular institution housing it is not designated, it should be assumed that the specimen housed in the institution where the author is known to have worked is the holotype, unless there is evidence that further material of the same gathering was used” (McNeill et al. 2006), was removed, the “syntype” label became correct; however, this can be stated as a fact now, especially since it is now known that another specimen of the original material was deposited at BM. In some databases, such as the NY Herbarium and JSTOR Global Plants, the status of the type had not been changed to “syntype” by the time of writing, because the label was still thought to have been attached by mistake.

**Figure 4.**
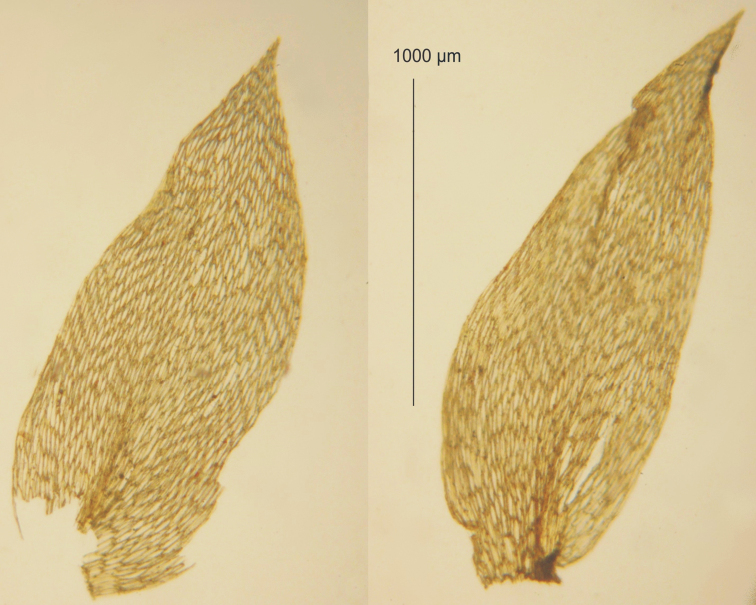
The best remains of top stem or branch leaves of *Stereodon nemoralis* Mitt. (NY 913349), loosely left in the envelope.

After careful analysis of the material, it can be said that the available material at NY (NY 913349) differs significantly from that which was photographed and published by Iwatsuki in 1970. It is easy to assess the changes in the amount of available material (Figs 2, 3). Our research confirms that the material from NY is preserved in very poor condition, and not as much turf is as visible as earlier (Figs 2, 3). The most important diagnostic characters of the species are not visible. In the case of *P. nemorale*, as well as the whole *Plagiothecium* genus, the most important taxonomic features are associated with stem leaves, i.e. their shape, symmetry and length, and with the shape, length and width of the middle part of the cells of these leaves. The examined specimen (NY 913349) does not have any whole stem leaves or leafy stems, and the available leaves are half or a third of normal size, suggesting that they originate from the top of the stem or from branches. Such leaves are not considered in the case of revision of the genus *Plagiothecium*. In addition, the leaves are considerably damaged: two of the best-preserved top stem or branch leaves are presented in Fig. 4. In its current state, the material indicates that the specimen belongs to the *Plagiothecium* genus, but there is no absolute certainty that it really belongs to *P. nemorale*. It should also be noted that the examined specimen (NY 913349) is not homogeneous: the fragments of the remains of the *Plagiothecium* specimen are accompanied by those of a young fern in the turf, which Mitten (1859) mentions in the diagnosis, as well as stems of other mosses (*Fissidens* sp.) (Fig. 3).

**Figure 5.**
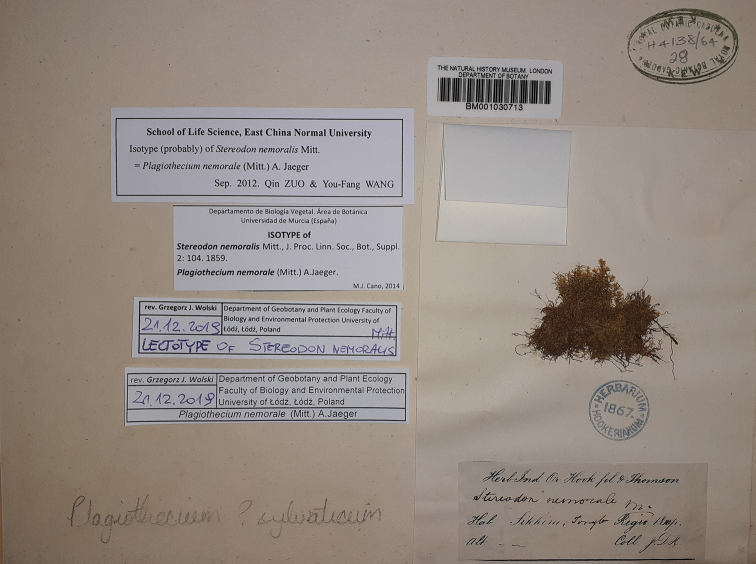
A specimen of *S. nemoralis* Mitt. from the BM Herbarium (BM 1030713).

As a part of the taxonomic revision of *P. nemorale*, efforts were made to find the entire original material and the species names now considered to be synonyms of this species. In the case of *S. nemoralis*, this also concerned specimens collected by Hooker from India or other materials from Mitten’s collections. A global search of herbaria revealed the existence of specimen BM 1030713, labelled as an isotype of *S. nemoralis* (Fig. 5), with a herbarium sheet bearing the stamp “Herbarium Hookerianum 1867”. We believe, however, that the date on this seal indicates the date of inclusion in Hooker’s herbarium at Kew (currently in BM), not the date of collection of the specimen.

This conclusion is supported by the following passage from Page 4 of Mitten’s book (1859): “The materials from the present enumeration have been derived from the collections (…), but more especially from those made by (…) Dr J.D. Hooker in the Sikkim-Himalaya and East Nepal (…). (…), the entire extensive collections of (…) Dr J.D. Hooker were entrusted to the author for segregation and distribution.”

In addition, Article 9.1 of the *Shenzhen Code* (Turland et al. 2018) states that “A holotype of a name of a species or infraspecific taxon is the one specimen or illustration (…) either (a) indicated by the author(s) as the nomenclatural type or (b) used by the author(s) when no type was indicated”. It would therefore appear that no holotype of *S. nemoralis* Mitt. exists, and that all the specimens, including that stored at the NY Herbarium (NY 913349), represent only a part of the material that was available to Mitten when he described the new species in 1859. According to Art. 9.6 (last sentence: “Reference to an entire gathering, or a part thereof, is considered citation of the included specimens”), these are syntypes.

Although the protologue by Mitten did not indicate the specimen or the illustration as a type according to Article 9.1 (a) of the *Shenzhen Code* (Turland et al. 2018), and merely cited a gathering by Hooker, the specimen from the NY Herbarium has nevertheless been considered a holotype for the past 50 years. For this consideration to be valid, the holotype would have to be the one specimen used by Mitten, according to Article 9.1 (b) of the *Shenzhen Code* (Turland et al. 2018); however, it is unclear whether the protologue only referred to the specimen now stored in the NY Herbarium, as this was not confirmed at the time of publication. The matter is further complicated by the existence of a duplicate specimen in the BM herbarium, as also not noted by Mitten (1859).

This specimen in NY has been considered as a holotype by a number of researchers (see: Iwatsuki 1970; Thiers 1992), and this information is still being reproduced in sources such as the NY Herbarium and JSTOR Global Plants (accessed on October 6, 2019). A detailed analysis was performed of the *Edinburgh Code* (Lanjouw et al. 1966) which was in force during Iwatsuki’s work, despite his paper being published only in 1970, and of the *Seattle Code*, which was in force in 1970 but was not yet published in print (Stafleu et al. 1972); the analysis highlighted three significant points: Article 7 Note 3 “If no holotype was indicated by the author who described a taxon, (...) a lectotype (…) for it may be designated”, Appendix III Point 1: “The choice made by the original author, if definitely expressed at the time of the original publication of the name of the taxon, is final. If he included only one element, that one must always be accepted as the holotype (…)”, and Appendix III Point 3: “A lectotype may be chosen only when an author failed to designate a holotype (...)”. Therefore, with this in mind, the specimen from NY (NY 913349) should be regarded as a lectotype rather than a holotype. However, in his revision for *S. nemoralis*, Iwatsuki (1970) cites only one specimen from the NY Herbarium, i.e. NY 913349, and recognises it as a holotype: this confirms that Iwatsuki was (probably) not aware of the existence of the additional original material of *S. nemorale* from the Mitten herbarium collected by J.D. Hooker in India, which was stored at the BM herbarium, or, perhaps, that it was rather a result of a “semi-mechanical” approach to the problem of typification of moss names.

Specimen NY 913349 cannot be recognised as a holotype based on Rec. 9A.4 of the *Vienna Code* posted above (McNeill et al. 2006), nor the *Tokyo Code* (Greuter et al. 1994), because it was not included in the *Edinburgh Code* (Lanjouw et al. 1966) or the *Seattle Code* (Stafleu et al. 1972) and could not be known to Iwatsuki in 1970; however, it could have been used at that time due to the author’s intuition and general knowledge of the matter. However, after being found to be in conflict with other provisions, Rec. 9A.4 was later removed from the *Melbourne Code* (McNeill et al. 2012).

In the protologue of *S. nemoralis*, not only did Mitten not refer to any single specimen, but also he explicitly stated that he was in possession of all materials acquired by J.D. Hooker and was responsible for their “segregation and distribution” (Mitten 1859). Considering this fact, and also Article 9.6 of the *Shenzhen Code* (Turland et al. 2018) stating that “A syntype is any specimen cited in the protologue when there is no holotype, or any one of two or more specimens simultaneously designated in the protologue as types” as well as Article 40.2 (Note 1) stating that “When the type is indicated by reference to an entire gathering, or a part thereof, that consists of more than one specimen, those specimens are syntypes”, it is obvious that both the specimens from the NY Herbarium (NY 913349), and the BM Herbarium (BM 1030713) are syntypes.

Due to the bad condition of the specimen from the NY Herbarium (NY 913349) and on the basis of the following five Articles of the *Shenzhen Code* (Turland et al. 2018), we propose that the specimen from the BM Herbarium (BM 1030713) be designated a lectotype of *S. nemoralis* Mitt.: Article 9.3, stating that: “A lectotype is one specimen or illustration designated from the original material (…) as the nomenclatural type, in conformity with Art. 9.11 and 9.12, if the name was published without a holotype, or if the holotype is lost or destroyed, or if a type is found to belong to more than one taxon”; Article 9.11 stating that: “If the name of a species or infraspecific taxon was published without a holotype (…), or when the holotype or previously designated lectotype has been lost or destroyed, or when the material designated as type is found to belong to more than one taxon, a lectotype or, if permissible (…), a neotype as a substitute for it may be designated”; Article 9.12: “In lectotype designation, an isotype must be chosen if such exists, or otherwise a syntype or isosyntype if such exists. If no isotype, syntype or isosyntype is extant, the lectotype must be chosen from among the paratypes if such exist. If none of the above specimens exists, the lectotype must be chosen from among the uncited specimens and cited and uncited illustrations that comprise the remaining original material, if such exist”; Article 7.10: “For purposes of priority (…), designation of a type is achieved only by effective publication (…)” and Article 7.11: “For the purposes of priority (…), designation of a type is achieved only if the type is definitely accepted as such by the typifying author, if the type element is clearly indicated by direct citation including the term ‘type’ (typus) or an equivalent, and, on or after 1 January 2001, if the typification statement includes the phrase ‘designated here’ (hic designatus) or an equivalent”.

## Description of the lectotype

**Figure 6.**
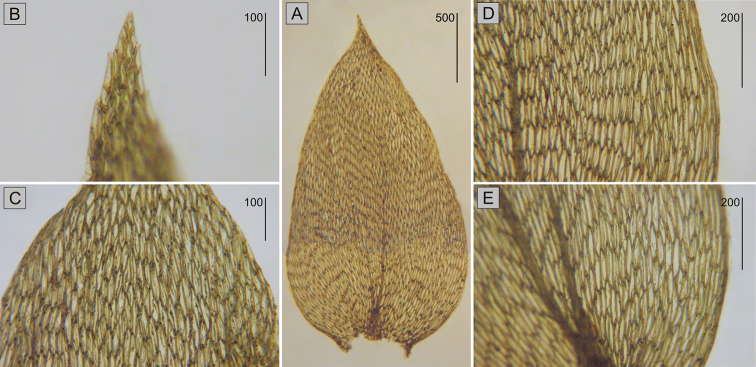
Lectotype of *S. nemoralis* Mitt. from the BM Herbarium (BM 1030713) **A** leaf shape **B** the serrated leaf apex **C–E** the shape and dimensions of cells from individual leaf zones: **C** from the upper part **D** from the middle part **E** from the lower part of the leaf. Scale bars: in µm.

The specimen from the BM Herbarium (BM 1030713) is a medium-sized plant, light green to yellowish, without metallic luster. Stems to 2 cm long, complanate-foliate, in cross-section rounded, with a diameter of 300–350 µm, central strand developed, epidermal cells 8–15 × 15–25 µm, parenchyma thin-walled, 20–50 × 11–35 µm; leaves gently concave, symmetrical, ovate, in dry condition shrunken, those from the middle of the stem 2.2–2.4 mm long, and the width measured at the widest point 1.0–1.3 mm; the apex straight, denticulate, acute, apiculate; two costae, extending almost to ½ leaf length, reaching 0.50–0.60 mm; hexagonal and narrowly-hexagonal cells in regular transverse rows, areolation very lax; cells reach 55–96 × 15–18 µm at the apex, 75–97 × 16–20 µm at mid-leaf, and 74–125 × 14–20 µm at the lower part of the leaf; decurrencies of 3 rows of rectangular cells, 25–35 × 20–30 µm (Fig. 6).

Throughout its range in Eurasia (Wolski 2017; Wolski and Nowicka-Krawczyk 2020) and North America (Wolski 2020), *P. nemorale* is characterised by: symmetrical leaves, a denticulate leaf apex, and decurrencies of two or three rows with mainly rectangular cells. Middle leaf cells are hexagonal to narrowly hexagonal, but their length does not exceed 100 µm. Thus, all the features of the described lectotype (BM 1030713) are within the range of variability for *P. nemorale* reported from the northern hemisphere (Wolski 2017, 2020; Wolski and Nowicka-Krawczyk 2020).

These features also distinguish very well *P. nemorale* from other similar or closely related species. For example, resurrected recently *P. longisetum* Lindb. is characterised by: asymmetrical leaves, a not denticulate leaf apex, and extended hexagonal leaf cells whose length is in the range from 100 to 150 µm (Wolski and Nowicka-Krawczyk 2020); *P. angusticellum* G. J. Wolski & P. Nowicka-Krawczyk is also distinguished from the species mentioned above by: an asymmetrical, but slightly curved and not denticulate apex, and narrowly elongate-hexagonal (113–143 × 15–19 µm), gently asymmetrical middle leaf cells (Wolski and Nowicka-Krawczyk 2020). *P. denticulatum* is distinguished from *P. nemorale* not only by leaf symmetry but mainly by decurrent angular cells rounded to rounded rectangular, inflated, forming very distinct auricles.

Formal typification may be summarised thus:

*Stereodon nemoralis* Mitt., Journ. Linn. Soc. Bot. Suppl. 1: 104 (1859) ≡ *Plagiothecium nemorale* (Mitt.) A. Jaeger, Ber. S. Gall. Naturw. Ges. 1876–1877: 451 (1878) ≡ P. silvaticum var. nemorale (Mitt.) Paris, Index Bryol.: 967 (1898). **Type citation**: *Hab.* In Himalayae orient. reg. temp., Sikkim, in monte Tonglo (ad radicem filicis cujusdam), *J. D. Hooker* ! **Lectotype** (*designated here*): “Herb. Ind Or Hook. Fil. & Thomson *Stereodon nemorale* m. Hab. Sikkim, Tonglo Regio temp. Alt. − J. D. H.” − BM 1030713!: **isolectotype**: NY 913349!
